# Structure–Function Relationship of Aminopeptidase P from *Pseudomonas aeruginosa*

**DOI:** 10.3389/fmicb.2017.02385

**Published:** 2017-12-05

**Authors:** Cui-Ting Peng, Li Liu, Chang-Cheng Li, Li-Hui He, Tao Li, Ya-Lin Shen, Chao Gao, Ning-Yu Wang, Yong Xia, Yi-Bo Zhu, Ying-Jie Song, Qian Lei, Luo-Ting Yu, Rui Bao

**Affiliations:** ^1^Pharmaceutical and Biological Engineering Department, School of Chemical Engineering, Sichuan University, Chengdu, China; ^2^Center of Infectious Diseases, State Key Laboratory of Biotherapy, West China Hospital, Sichuan University and Collaborative Innovation Center, Chengdu, China; ^3^School of Life Sciences and Engineering, Southwest Jiaotong University, Chengdu, China

**Keywords:** *Pseudomonas aeruginosa*, aminopeptidase P, virulence, tri-nuclear form, X-ray crystallography

## Abstract

*PepP* is a virulence-associated gene in *Pseudomonas aeruginosa*, making it an attractive target for anti*-P. aeruginosa* drug development. The encoded protein, aminopeptidases P (Pa-PepP), is a type of X-prolyl peptidase that possesses diverse biological functions. The crystal structure verified its canonical pita-bread fold and functional tetrameric assembly, and the functional studies measured the influences of different metal ions on the activity. A trimetal manganese cluster was observed at the active site, elucidating the mechanism of inhibition by metal ions. Additionally, a loop extending from the active site appeared to be important for specific large-substrate binding. Based on the structural comparison and bacterial invasion assays, we showed that this non-conserved surface loop was critical for *P. aeruginosa* virulence. Taken together, these findings can extend our understanding of the catalytic mechanism and virulence-related functions of Pa-PepP and provide a solid foundation for the design of specific inhibitors against pathogenic-bacterial infections.

## Introduction

*Pseudomonas aeruginosa* is a common nosocomial pathogen and is notoriously difficult to treat due to its high intrinsic and acquired drug resistance (Hancock, 1998; Stover et al., 2000). To address the challenges of *P. aeruginosa* infections, alternative approaches other than conventional antibiotic therapy were undertaken, with an emphasis on anti-virulence strategies (Hentzer et al., 2003; Luckett et al., 2012; Gi et al., 2014; Hwang et al., 2016; Maura et al., 2016; Johnson and Abramovitch, 2017; Rampioni et al., 2017). The targets for most anti-virulence strategies are those well-studied virulence factors (adhesins, toxins, effector secretion system components) that directly participate in pathogen-host cell interactions (Woods et al., 1982; Hahn, 1997; Hood et al., 2010). However, not all of these virulence factors play significant roles in *P. aeruginosa* infection (Miyata et al., 2003). Instead, there are many genes with unidentified roles in *P. aeruginosa* virulence, while the corresponding mutants resulted in obvious virulence attenuation, suggesting potential applications for these genes in anti-virulence therapy discovery.

*PepP* encodes an enzyme belonging to the aminopeptidases P (APPro) family (E.C.3.4.11.9), a type of metalloprotease that catalyzes the removal of the N-terminal residue from a polypeptide that has proline as the second residue (Taylor, 1993; Gonzales and Robert-Baudouy, 1996; Wilce et al., 1998). It is one of the critical virulence-associated genes identified using a *P. aeruginosa*-*C. elegans* infection mode (Feinbaum et al., 2012). In this model, transposon mutants of *pepP* in *P. aeruginosa* PA14 have attenuated virulence, leading to reduced *C. elegans* survival. *PepP* is highly conserved in all *P. aeruginosa* genomes sequenced to date and with high similarity to this gene from other *Pseudomonas* species (82.4%–100% identity). In contrast to other metalloaminopeptidases, APPro is a cytoplasmic aminopeptidase and recognizes a restricted scissile Xaa-Pro bond of those polypeptide or protein substrates that may be linked to virulence-associated phenotypes or other biological processes beyond the general protein degradation ability (Lowther and Matthews, 2002). APPro is widely distributed among bacteria, fungi, plants and mammals. It is thought to play a role in many important biological pathways, including hormone regulation in mammals (Simmons and Orawski, 1992) and the terminal degradation of proline-containing peptides and proteins (Wilce et al., 1998), or organophosphate compounds in bacterial (Jao et al., 2004). The gene coding *Escherichia coli* aminopeptidases P (Ec-PepP) was even identified as a factor involved in outer membrane vesicles (OMV) production (McBroom et al., 2006; Vanaja et al., 2016). While structural and biochemical studies on APPro have visualized a general catalytic mechanism and revealed a conserved binding pocket for N-terminal substrates (Wilce et al., 1998; Graham et al., 2005, 2006; Liu et al., 2007; Graham and Guss, 2008; Jeyakanthan et al., 2009; Weaver et al., 2014), further investigations are needed to elucidate the structural basis for APPro’s specific substrate recognition and diverse functions. Thus, a better understanding of the structure and function of *P. aeruginosa* APPro (Pa-PepP) would enable us to propose the possible mechanisms involved in bacterial virulence.

In this study, we solved the X-ray crystal structure of Pa-PepP with a resolution of 1.8 Å. A biochemical analysis verified the residues critical for catalysis. The presence of a trinuclear manganese cluster in the reaction center suggests a mechanism for inhibition by excessive metal ion binding. Furthermore, an extended substrate binding site was identified to be responsible for virulence-related protein recognition.

## Results

### Pa-PepP Adopts a Canonical Pita-Bread Fold and Assembles as a Tetramer in Crystal

**FIGURE 1:**
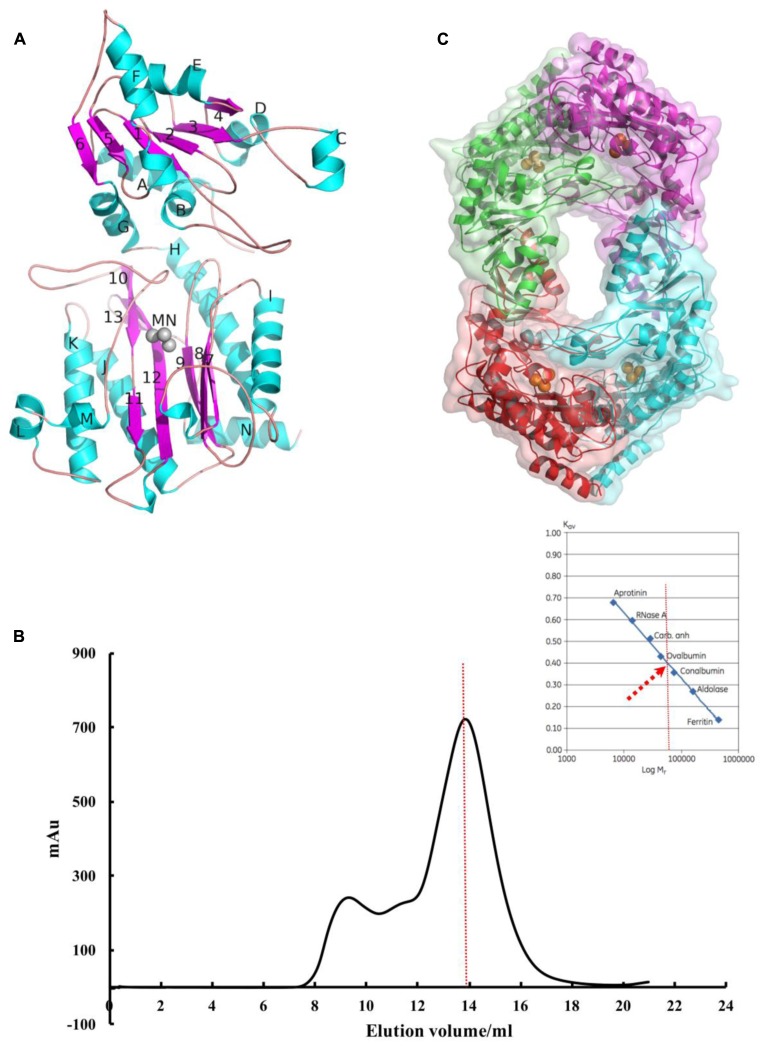
Pa-PepP adopts a canonical pita-bread fold and assembles as a tetramer in crystal. **(A)** The second structure assignments of the Pa-PepP monomer. Beta-sheets are colored blue, alpha-helices are colored green, and the three Mn^2+^ ions are shown as gray spheres. The figure was generated by PyMOL (http://www.pymol.org). **(B)** The gel filtration curve for the purified recombinant Pa-PepP in solution. The AUC results are presented as the ultraviolet absorption in 280 nm. The black solid curve refers to Pa-PepP eluted from GE HiLoad 16/600 Superdex 200 column. The red dash curve refers to the sample elution volume and corresponding molecule mass of the sample when compared with the standard sample proteins. **(C)** 3D structure of Pa-PepP showed a detailed tetramer of four subunits or a homodimer of Pa-PepP. Each subunit is presented as different color with Mn^2+^ ions are shown as orange spheres in the metal binding site.

Refinement of the Pa-PepP structure resulted in a final model with a free R-factor (Rfree) of 0.2206 at 1.8 Å. Sufficient electron density allowed us to model all residues from 1 to 444. The crystallographic statistics are summarized in **Table 1**. The monomer structure displays a two-domain organization where the N-domain (1–175) is composed of a mainly parallel beta-sheet core (B1–B6) flanked by seven alpha helices (A–G). In contrast, the catalytic C- domain (176–444) adopts a conserved “pita-bread” fold with six beta sheets (B7-B12) in antiparallel configurations (**Figure 1A**). This pita-bread fold is commonly found in N-terminal amido-, imido-, and amidino-scissile bond-cleaving enzymes, and serves as a structural basis for the metal-dependent catalysis (Lowther and Matthews, 2000; Besio et al., 2010).

**Table 1:** Data collection and refinement statistics.

	PDB Entry	5WZE
**Data collection**	Space group	P 21 21 21
	**Cell dimension**	
	*a, b, c (Å)*	111.197,123.432,149.485
	α, β, γ (°)	90, 90, 90
	Wavelength	0.97776
	Resolution (Å)	48.01–1.783 (1.847–1.783)
	I/I	19.714 (2.167)
	Completeness (%)	0.99
	*R*_merge_	0.099 (0.709)
	Redundancy	8.9 (6.4)
**Refinement**	Resolution (Å)	48.01–1.783 (1.847–1.783)
	No. of reflections	194404 (18600)
	Rwork/Rfree	0.2105/0.2206
	**No. of atoms**	
	Protein	1783
	Ligand/ion	90
	Water	1153
	**B-factor**	
	Protein	27.37
	Ligand/ion	38.94
	Water	30.89
	***R.m.s. deviations***	
	Bond lengths (Å)	0.011
	Bond angles (°)	1.00
	Ramachandran plot	98/1.5/0

Although the recombinant Pa-PepP purified from *E. coli* strain BL21 (DE3) exists as a monomer in solution (**Figure 1B**), tetrameric oligomerization was observed in crystal packing (**Figure 1C**). The monomers were arranged as a dimer with an extended loop contributing to the active site of the adjacent subunit and an average interface area of approximately 2050.9 A^2^ per subunit. The dimer-of-dimers was generated by crystallographic symmetry operation (x–1/2, –y–1/2, –z), and the major interactions are contributed by the C-domain of each monomer, resulting in an ∼815.6 A^2^ buried area per subunit. For most metalloaminopeptidases, the oligomeric state is essential for their biological functions. The loop from the adjacent monomer extends the substrate-binding site and thus enables the enzyme to cleave larger substrates. Thus, the antiparallel dimerization would be the basic active polymerization forms to its biological activity, and the different quaternary arrangement may reflect a possible regulatory mechanism for its activity.

### Effect of the Metal Ion in Activity Modulation

**FIGURE 2:**
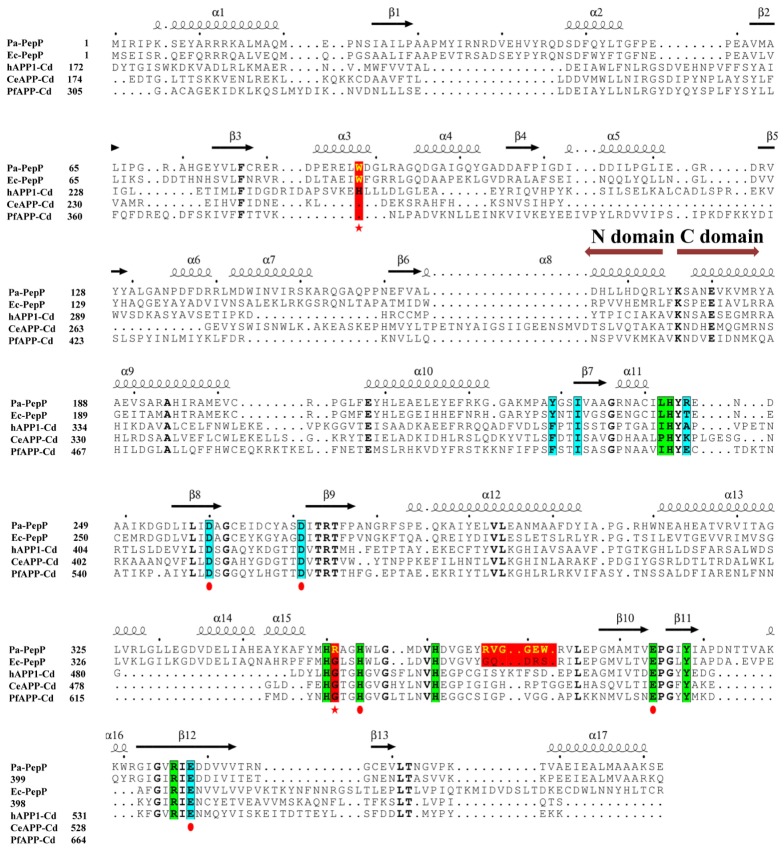
Multiple sequence alignment of the truncated N domain and catalytic C domains from X-prolyl peptidases. From top to bottom, the sequences are aminopeptidases P from *P. aeruginosa* (Pa-PepP), aminopeptidases P from *Escherichia coli* (Ec-PepP), truncated N domain and catalytic C domains from from human XPNPEP1 (hAPP1, 172-618), X-prolyl aminopeptidase from *Caenorhabditis elegans* (CeAPP, 174–616), and aminopeptidase P from *Plasmodium falciparum* (PfAPP, 305–774). Residues for chelating metal ion are highlighted using the red circle under the sequences; Residues contributed to S1 pockets are highlighted with a blue background and residues for S’ sites are presented with a green background; The red color displayed residues or short sequences are non-conserved among prokaryotic and eukaryotic APPros, which are also proved to be important to APPros’ function. Sequences were aligned using the program ClustalX (Li et al., 2008), and the alignment was presented using the online ESPript 3.0 server (http://espript.ibcp.fr/ESPript/ESPript/).

**FIGURE 3:**
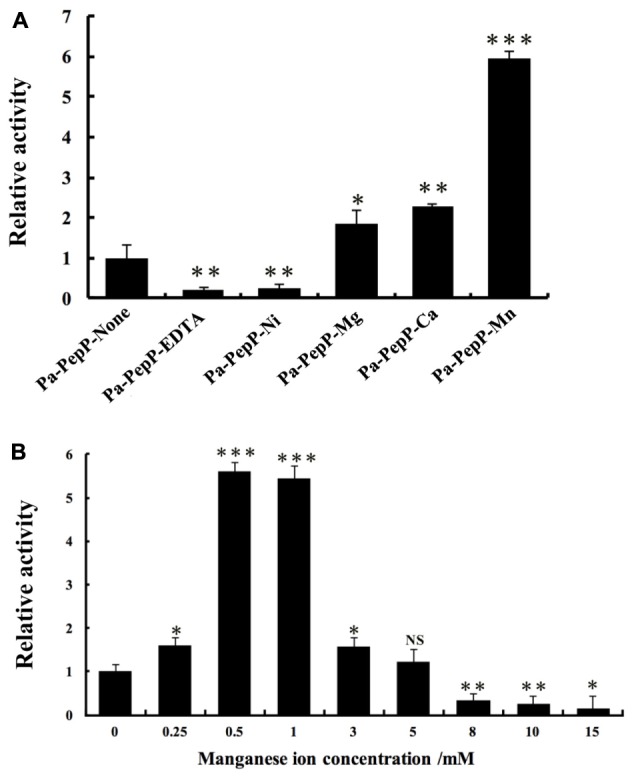
Relative activity assay of Pa-PepP using the quenched fluorescent substrate Lys (Abz)–Pro–Pro–pNA. All assays were performed at 37°C for 5 min in the presence of 50 mM Tris (pH 8.5), 100 mM NaCl, 0–250 μM Lyz(Abz)–Pro–Pro–pNA quenched fluorescent substrate, and 1 μg/mL^-1^ Pa-PepP that had been incubated with different metal ions at 37°C for 10 min. Each bar represents the mean of three independent measurements (SEM). **(A)** Relative activity of Pa-PepP in the presence of 1 mM different metal ions or EDTA. **(B)** Relative activity of Pa-PepP in presence of different amount of Mn^2+^. *P*-values for comparison of each group with Pa-PepP-alone **(A)** or 0 mM Mn^2+^ **(B)** were determined by 2-tailed Student’s *t*-test (^∗^*P* < 0.05; ^∗∗^*P* < 0.01; ^∗∗∗^*P* < 0.001).

Metalloaminopeptidases are well known for their metal-dependent catalysis mechanisms. Proline-specific aminopeptidases such as APPro and prolidase prefer dinuclear Mn(II) cluster as cofactor (Wilce et al., 1998; Graham et al., 2005). The residues coordinating the two Mn atoms (Mn_A_ and Mn_B_) are highly conserved among APPro family members (**Figure 2**), and the non-equivalent roles of the two metal ions in catalysis have been extensively studied (Graham et al., 2005; Hu et al., 2007). To test the role of divalent metal cluster on Pa-PepP, we measured the relative activities of Pa-PepP in the presence of 1 mM Mn^2+^,Ca^2+^,Mg^2+^, Ni^2+^, Zn^2+^, and EDTA (**Figure 3A**). The addition of Mn^2+^ significantly restored the Pa-PepP activity, while the limited enhancement of activity was observed upon addition of Ca^2+^ or Mg^2+^. The exhibited basal activity may be a result of the partially pre-bound metal ions from the cell, and the limited effects of Ca^2+^ and Mg^2+^ may be due to their weak binding affinity to the active site. Previous studies on metal selection in Ec-PepP revealed that the Zn^2+^ ion has high affinity for APPro and inhibits the hydrolysis reaction by occupying a third metal binding site (Graham et al., 2005; Hu et al., 2007). Although we could not measure the effect of Zn^2+^ ion on Pa-PepP because of the formation of zinc hydroxide precipitates upon adding ZnCl_2_ to the alkaline enzyme buffer, we observed the complete inhibition of enzyme activity in the presence of both the Ni^2+^ ion and the chelating agent EDTA. These results suggested that the Ni^2+^ may also possess high affinity to APPro and tends to bind to those positions equivalent to Zn^2+^ ions in Ec-PepP.

**FIGURE 4:**
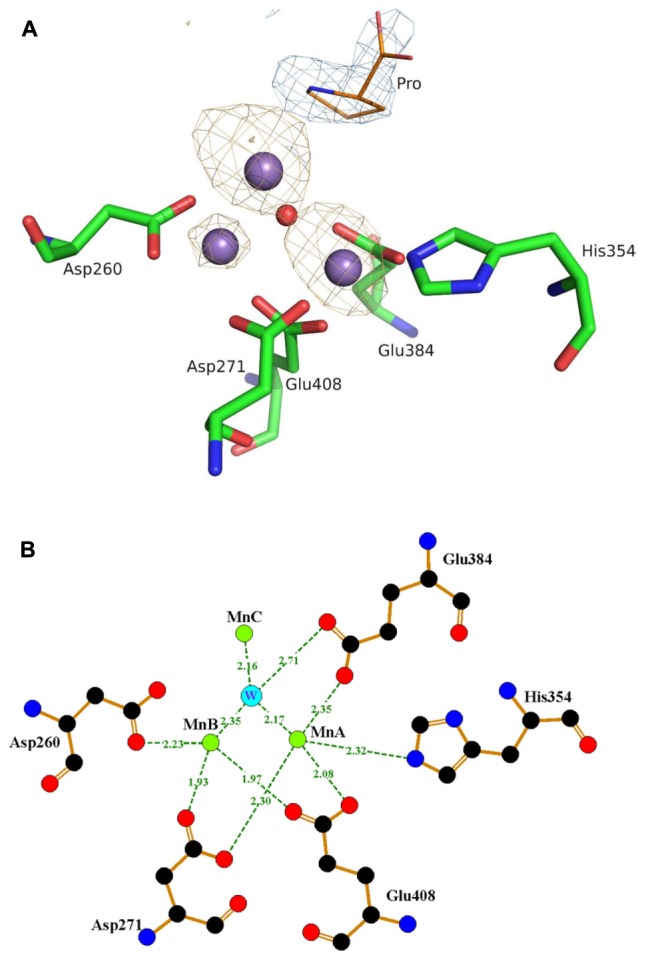
The trimetal Mn cluster center and interactions with the surrounding amino acid residues. **(A)** The anomalous map peaks of the bound Mn in Pa-PepP structure. The three Mn^2+^ ions are shown as dark purple spheres with different anomalous signals and the red sphere refers to the water molecule participated in activity modulation. For clarity, only part of the metal binding residues is shown together with the observed proline with blue electron density near the active site. The figure was generated by PyMOL (http://www.pymol.org). **(B)** Representative interactions around the active sites. Mn_A_ is liganded by the side chains of His354, Asp271, Glu384, Glu408, and Mn_B_ interacts with Asp260, Asp271, Glu408. And Mn_C_ interacts through one solvent molecular (W) to Mn_A_ and Mn_B_ with a metal separation distance of 3.1–3.7 Å. Figures were prepared using LIGPLOT (Wallace et al., 1995).

To identify the binding state of Mn^2+^ ions in active site, 10 mM MnCl_2_ was added during the crystallization experiment, along with 10 mM proline. In the final structure, there are three Mn atoms in each monomer (**Figure 4**). Mn_A_ is liganded by the side chains of His354, Asp271, Glu384, Glu408, and Mn_B_ interacts with Asp260, Asp271, Glu408. These interactions are highly conserved among those reported APPro structures that resulted in a distorted trigonal-pyramidal coordination network. The smaller anomalous signals and 0.5 occupancies of the Mn_B_ site confirmed one of the structural features in metal-dependent aminopeptidase: the metal binding site B has relatively weaker binding affinity comparing to the site A (Hu et al., 2007). Additionally, the third Mn atom (Mn_C_) is located in a position previously identified as being occupied by water in Zn-load Ec-PepP structures (Graham et al., 2005). The strong anomalous peak at this position verified its identity as a tightly bound Mn^2+^ ion rather than a water molecule. It is interesting to note that this Mn_C_ position is different from the third metal binding site found in Ec-PepP, in which the Zn_C_ is coordinated by His243, His361, and the hydrolysis product Pro residue (Graham et al., 2005). In Pa-PepP, Mn_C_ interacts through one solvent molecular (W) with Mn_A_ and Mn_B_, with a metal-separation distance of 3.1–3.7 Å. The Pro residue lies immediately above the solvent molecular W but does not participate in the interaction with Mn_C_. Remarkably, Mn_C_ occupies the S1 subsite that ordinarily binds the substrate, providing an explanation for the inhibition effect of excessive metal binding. To verify the inhibitory effect of Mn^2+^ ion, we performed an activity assay in the presence of different amounts of Mn^2+^. The result is consistent with our structural analysis (**Figure 3B**): there is a Mn^2+^ ion concentration-dependent activity regulation pattern, in which 0.5–1 mM of Mn ion is the optimal concentration for 20 nM (1 μg/ml) Pa-PepP, and inhibitory effects are observed when Mn^2+^ ion exceeded 5 mM. Inhibition was not observed at ion concentrations up to 20 mM in the assays with Ca^2+^ and Mg^2+^ (**Supplementary Figure S1**).

### Conserved Active-Site Motif and Extended Binding Site for Specific Substrate Recognition

**FIGURE 5:**
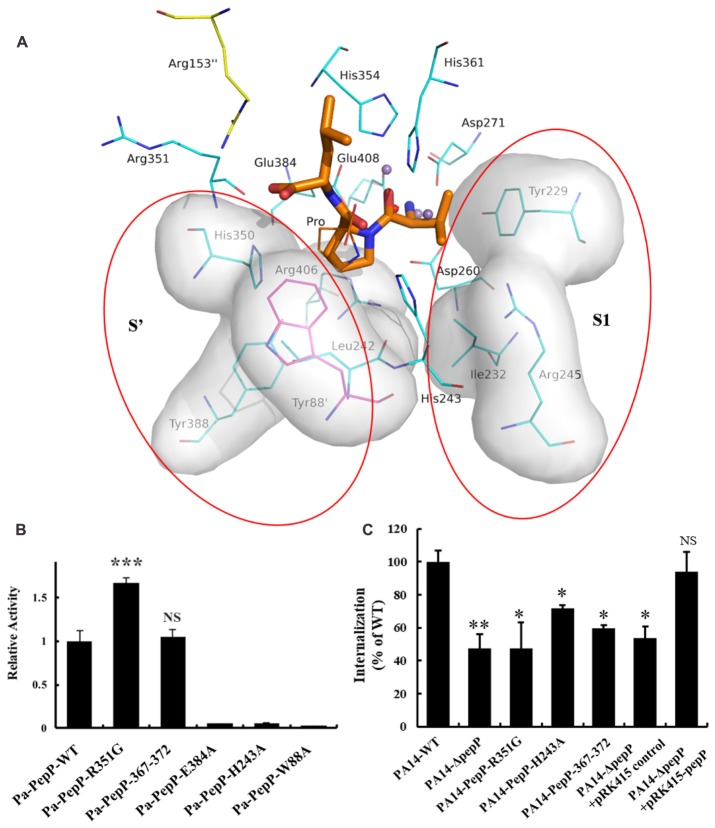
The modeled Val-Pro-Leu bound Pa-PepP complex and the important residues or sequences identified to be important to Pa-PepP enzyme activity and bacterium virulence. **(A)** The modeled Val-Pro-Leu bound Pa-PepP complex by superposing with the substrate bound Ec-PepP structure (PDB code: 2BN7). The left and right shadow highlighted with red circles refers to the predicted substrate binding sites (S’ and S1 sites) containing these mainly related residues, with Trp88 from the adjacent subunit colored magenta. Val-Pro-Leu is colored orange for the main carbon chain with thick lines. Other residues for direct interactions to metal ions are colored gray. **(B)** Relative activity of wild-type and mutant Pa-PepP. Activity was measured using the quenched fluorescent substrate Lys(Abz)–Pro–Pro–pNA as mentioned before. Each bar represents the mean of three independent measurements (±SEM). **(C)** Bacterial invasion of HeLa cells upon 1 h of infection at an MOI of 10 with *P. aeruginosa* strains grown at the transition between the exponential and stationary phases in LB broth at 37°C. Each bar represents the mean of three independent measurements (±SEM). *P*-values for comparison of each group with Pa-PepP-WT were determined by 2-tailed Student’s *t*-test (^∗^*P* < 0.05; ^∗∗^*P* < 0.01; ^∗∗∗^*P* < 0.001).

Several APPro structures have been reported to date, including APPro from *Escherichia coli* (Ec-PepP, 1M35) (Wilce et al., 1998), *Streptococcus thermophilus* (3IL0), *Bacillus anthracis* (3IG4), *Yersinia pestis* (4PV4), *Thermotoga maritima* (2ZSG), *Streptococcus pyogenes* (3OVK), human (hAPP1, 3CTZ) (Li et al., 2008), *Caenorhabditis elegans* (CeAPP1, 4S2R) (Iyer et al., 2015) and *Plasmodium falciparum* (PfAPP, 5JQK) (Drinkwater et al., 2016). Ec-PepP shares the highest sequence identity with Pa-PepP and both of them are prokaryotic APPro (Gonzales and Robert-Baudouy, 1996). All of these APPros share a common catalytic domain structure that contains a metal center flanked by the S1’-S1 pockets (**Figures 2, 5A**). Previous structural studies on Ec-PepP in complex with the substrate or inhibitor have revealed those pockets (Graham et al., 2004; Graham and Guss, 2008). We modeled the Val-Pro-Leu bound Pa-PepP complex by superposing Pa-PepP structure with the substrate-bound Ec-PepP structure (PDB code: 2BN7) (**Figure 5A**). The shallow hydrophobic S1’ pocket could be defined by the pre-bound Pro residue, Arg406, Tyr388, and His350. S1’ sites are conserved in APPro and prolidase but not in other metalloaminopeptidases, which implies that they play critical roles in regulating the activity of proline-specific peptidases. Additionally, in prokaryotic APPros and prolidases, a tryptophan (Trp88’ in Pa-PepP) from an adjacent subunit extends the boundary of S1’ site and contributes to the P1’ Pro binding (Yoshimoto et al., 1994; Jao et al., 2006). At the S1’-S1 junction, His243 and His361 interact with the main-chain carbonyl of P1’ Pro and P1 Val, respectively, indicating their specific roles in recognizing and stabilizing the stereospecific scissile X-Pro bond. In the S1 pocket, the side chain of P1 Val faces Tyr229, Ile232, Arg245, while its main chain replaces the Mn_C_ ion and is involved in the Mn_A_ and Mn_B_ ion mediated interactions. In metallomainopeptidases, the dinuclear Mn center is thought to be essential for nucleophilic attack (Graham et al., 2005). To verify the impacts of the critical motifs revealed by structural analysis, we constructed Ala substitutions on those motifs (Trp88 for S1’ pocket, His243 for scissile bond stabilizing, Glu384 for metal binding) and verified that the expression level and stability are comparable between the wild type and all the mutants (**Supplementary Figure S2**). The Pa-PepP activity assay shows that all the mutations would eliminate enzymatic activity, which further confirmed that the evolutionary conservation in those regions is strongly associated with the essential functional roles of APPro family (**Figure 5B**).

In contrast to the prolidases that cleave dipeptides only, APPro is capable of modifying protein substrates. The relatively larger substrate binding ability of APPro is partly due to the extension of the substrate binding site beyond the S1’–S1 region (Drinkwater et al., 2016). The modeled tripeptide-bound Pa-PepP structure indicated that Arg153 and Arg351 accommodate the carboxylate group of the P2’ Leu, and guide the C-terminus of the substrate toward the tetramer surface (**Figure 5A**). Furthermore, the R351G mutation results in an increase in enzyme activity. The similar Arg153/Arg370 basic patch is also found in Ec-PepP, in which the non-basic residue substitution on either of them leads to an obvious decrease in *K_m_* and an increase in *k_cat_/K_m_* (Jao et al., 2006). Thus, this basic patch, which is located at the entrance of catalytic cave, can play an important role in specificities of protein substrate recognition and orientation.

### Structural Analysis Identified a Surface Loop Participating in *P. aeruginosa* Virulence

The conserved structural properties in active site suggest a common catalytic mechanism for APPro family members, while the differences in the substrate-binding regions indicate the specific functions for each APPro member. Next to the P2’ binding site, a surface loop (residue 365–377 in Pa-PepP) between helix α_15_ and sheet β_10_ was identified as a candidate for specific functions due to the diversity in its sequences between APPro members (**Figure 2**). To investigate its impact on enzyme catalytic activity, we mutated residues RVGGEW (Pa-PepP 367-372) to GQDRS (Ec-PepP 368-372). Equivalent activities were observed between the corresponding mutant and the wild-type (**Figure 5B**). Since, in the activity assay, we used a non-specific tripeptide analog instead of the native substrates, these results may not accurately reflect the actual substrate-binding situation.

To further elucidate the impacts of Pa-PepP mutations on the bacterium virulence, three mutants with different effects on enzymatic activity were made in *P. aeruginos* PA14 for use in a Hela-cell invasion assay (**Figure 5C**). The PA14 bacterial growth *in vitro* was not affected by *pepP* deletion or any mutations in *pepP* (data not shown). The results showed that Pa-PepP knockout PA14 (PA14-Δ*pepP*) strain lost half of its ability to enter mammalian cells compared to wild-type PA14, and the cell invasion ability for PA14-Δ*pepP* strain was restored when introducing a complementary plasmid pRK415 for Pa-PepP expression, which confirmed the contribution of Pa-PepP to *P. aeruginos* virulence (Feinbaum et al., 2012). The enzymatic inactive mutant (H243A) exhibited an attenuated invasion ability, suggesting a role for the cleavage activity of Pa-PepP in its biological function. Strikingly, the PA14-R351G mutant also displayed weak cell internalization efficiency comparable to PA14-Δ*pepP*, revealing that the specificity of protein-substrate recognition is indispensable to the virulence-related function. The mutant PA14-Pa-PepP-367-372 also presented an attenuated invasion ability, verifying the critical role of 367-372 loop in the biological function of Pa-PepP, even though it does not have an obvious impact on enzymatic activity.

## Discussion

Aminopeptidases hydrolyze the N-terminal residues of peptide/protein substrate and generally have broad specificity. They are associated with various essential biological functions and have been suggested as suitable therapeutic targets for corresponding diseases (Griffith et al., 1997; Pasqualini et al., 2000; Gardiner et al., 2006; Stack et al., 2007; Trenholme et al., 2010; Skinner-Adams et al., 2010). There are a number of aminopeptidases inhibitors that have already been developed as potent drug candidates (Prechel et al., 1995; Maggiora et al., 1999; Aozuka et al., 2004; Krige et al., 2008). Given the rare occurrence of proline as the second residue in protein, the proline-specific APPro exhibits even narrower substrate specificities, making it an attractive target for specific therapeutic application. In Feinbaum et al.’s (2012) *C. elegans* model, aminopeptidase PepP gene was identified as a virulence related factor and was involved in the highly attenuated *C. elegans* deaths, which suggested that Pa-PepP may be a potential drug target for the treatment of *P. aeruginosa* infections. However, other infection models are still needed to further confirm the role of *pepP* in *P. aeruginosa* virulence, and mechanisms account for Pa-PepP’s correlation with bacterium virulence remain unknown. In the *C. elegans* model, the PA14 strain with transposon insertions in gene *pepP* display increased pyocyanin levels, whereas pyocyanin has been demonstrated to play an important role in *P. aeruginosa* virulence in many models of infections (Lau et al., 2004; Dietrich et al., 2006). Thus, we predict that there may be other mechanisms linked to the effect of *pepP* on the virulence associated phenotypes. In this work, we analyzed the specific substrate binding site of Pa-PepP based on structural studies, as well as its consensus signatures and mechanisms, which provided clues for better understanding its unique physiologic function.

Like all other metalloaminopeptidase, APPro requires metal ion for its enzyme activity, and the manganese might be the most preferred cation. The dinuclear metal center structure was consistent among all APPro members, with the M_B_ ion binds more loosely than M_A_. Different anomalous map peaks of the bound Mn in Pa-PepP structure verified the inequivalent occupancy of two metal-binding sites. It should be noted that the different binding affinities of the metal sites allow for the sequential addition of multiple types of metal ions in other metalloaminopeptidases, resulting in diverse enzymatic activities (Carpenter and Vahl, 1973; Van Wart and Lin, 1981; Allen et al., 1983; Bayliss and Prescott, 1986; Lin and Van Wart, 1988). Stimulatory or inhibitory effects of various divalent metal ions were also observed in both Ec-PepP and Pa-PepP, suggesting that the metal selectivity contributes to activity-regulating mechanisms in APPro. Furthermore, the additional M_C_ atom found in Pa-PepP provides a structural basis for the manganese concentration-dependent modulation of activity. In conclusion, the delicate structure of the metal binding sites enables the APPro to precisely sense the type and concentration of metal ions, facilitating its scalability in specific function performances.

The individual domain superposition of the APPros shows a close agreement between the catalytic domain structures (rmsd values of 0.847–1.846) and the nearly identical active sites allow them to maintain the unique catalytic mechanism. However, the extensive sequence diversity between prokaryotic and eukaryotic APPros, especially with respect to the domain assignments, domain motions, and oligomeric assemblies, are the breakthrough points for understanding the particular functions of each member (**Supplementary Figure S3**). For instance, the antiparallel dimerization observed in prokaryotic APPro resulted in an inward extending loop region that reached into the cavity of the neighboring subunits and participated in the P1’ residue binding. Instead, in eukaryotic species, this part was replaced by the additional N-domain to ensure the maintenance of the active site pocket (Li et al., 2008). Moreover, those different oligomerization interfaces may affect the substrate selectivity in APPros.

It has been proposed that the tetrameric assembly is indispensable for Ec-PepP’s function because the increase of the binding surface area can accommodate larger protein substrates (Yoshimoto et al., 1994). Pa-PepP possesses more positively charged groups on its solvent-accessible area compared to Ec-PepP (**Supplementary Figure S4**), implicating a preference for substrate selection. Notably, the 367:372 loop identified from Pa-PepP is located at the entrance region of the catalytic cave and replacement of this loop by corresponding fragment from Ec-PepP attenuated the cell invasion ability of PA14. These observations revealed that residue variations and charge distribution differences around the substrate binding interfaces can lead to distinct functions for Pa-PepP and Ec-PepP, even though they shared high sequence similarity.

## Conclusion

APPros exert specific physiologic functions, and many of them are relate to virulence in bacteria. However, due to the insufficiency of information about its biological substrate, the knowledge of its biology functions are limited for now. Here, we discussed the ion modulation mechanism in Pa-PepP and unveiled the differences in the regions surrounding substrate binding site, which extend our understanding about the catalytic mechanism and virulence-related functions of Pa-PepP. Most importantly, the structural analysis provides us a solid foundation for designing specific inhibitors against pathogenic bacterial infection by blocking the particular protein substrate binding site instead of directly interfering the common catalytic center.

## Materials and Methods

### Protein Expression and Purification

Full-length *pepP* was amplified by PCR using gene-specific primers (Supplementary Table S1) from the *P. aeruginosa* PA14 genome DNA on Gene amplification Machine (Gene Touch, BIOER, HangZhou, China). Full-length *pepP* containing six C-terminal histidine residues (LEHHHHHH) was homologous recombined with the linearized pET-22b (+) using a ClonExpressTM II One Step Cloning Kit (Vazyme). All point mutants were generated using the QuickChange (I-5TM2^∗^High Fidelity Master Mix, MCLAB) PCR-based method, on the pET-22b (+) construct. The recombinant plasmid was transformed into *E*. *coli* strain BL21 (DE3) for protein expression. The bacterial culture was grown in LB medium in the presence of 100 μg mL^-1^ ampicillin and incubated with shaking at 310 K until the OD600 reached 0.9 (ZhiChu, ShangHai). The culture was cooled to 289 K before protein expression was induced with 0.2 mM IPTG for 20 h (Bao et al., 2013). Following induction, the bacteria were collected and resuspended in a lysis buffer consisting of 25 mM Tris–HCl pH 8.5, 10 mM NaCl, 5 % glycerol and 1 mM phenylmethanesulfonyl fluoride (20 g of cells/100 mL buffer, Sigma–Aldrich) and lysed by sonication. The lysate was cleared by centrifugation at 11000 *g* for 45 min and then the supernatant was loaded onto a 2 mL Ni–NTA affinity resin (Qiagen) for 2 L culture. The Ni–NTA column was washed with ten column volumes of the lysis buffer supplemented with 20 mM imidazole. The target protein was eluted with the same buffer in the presence of 200 mM imidazole. Fractions were pooled and determined by SDS–PAGE, followed by further purification on size-exclusion chromatography Superdex 200 column (GE Healthcare), which was pre-equilibrated with the buffer consisting of 25 mM Tris–HCl pH 8.5, 10 mM NaCl. Fractions containing Pa-PepP were pooled and concentrated to a concentration of approximately 16 mg mL^-1^ using a Centricon filter (10 kDa cutoff; Millipore, Billerica).

### Crystallization and Data Collection

Initial crystallization experiments were carried out using four commercial crystallization screens from Hampton Research and Rigaku (Index HT, Crystal Screen HT, WIZARD HT, XTAL QUEST HT). Crystallization screens were conducted as previously described with some modifications (Bao et al., 2009). Briefly, crystallization initially screens were carried out using a Mosquito liquid dispenser by hanging-drop vapor-diffusion method at 291 K in 96-well plates. The 200 nL mixing drop containing protein solution and reservoir buffer by 1:1, with a final protein concentration of 16 mg ml^-1^ in 25 mM Tris–HCl pH 8.5, 10 mM NaCl. The final optimized crystals for the recombinant protein were obtained by mixing 2 μl of the protein sample containing Mn^2+^ and proline with an equal volume of the reservoir solution containing 30% PEG400, 100 mM sodium cacodylate pH 6.5, 200 mM lithium sulfate. Crystals grew in approximately 2–3 days and were transferred to a cryo-protectant solution (reservoir solution with 6% PEG400) prior to flash-cooling in liquid nitrogen. X-ray data were collected with a CCD camera on BL-17U stations of the SSRF, China. The diffraction data were indexed, integrated, and scaled using the HKL2000 program suite (Otwinowski and Minor, 1997).

### Structure Determination and Refinement

Data processing and scaling were carried out using the HKL2000 software package. The data were processed to a resolution limit of 1.847–1.783 Å (Rmerge = 0.099) in space group P2, with unit-cell parameters *a* = 111.197, *b* = 123.432, *c* = 149.485 Å. The phase problem was solved by molecular replacement using PHENIX with aminopeptidase P from *E. coli* (PDB entry 1az9; space group P 6422; resolution 1.9 Å) as a template. The process of structure building and refinement was monitored using the COOT (Emsley et al., 2010). Water molecules were automatically added by PHENIX (Adams et al., 2010).

### Differential Scanning Calorimetry (DSC) Assays

Differential scanning calorimetry (DSC) is a promising thermoanalytical technique used for evaluating the stability of proteins as well as other biomolecules. The enthalpy value determined by DSC can provide direct information about the energetics of thermally induced processes, and the measured melting temperature(Tm) reflects thermal stability of proteins. The assays were conducted on the MicroCal VP-Capillary DSC System (Malvern). Protein samples were prepared with a final concentration of 1 mg/ml in 400 μl buffer and each sample has another 400 μl blank buffer as reference which were loaded in pairs. Operating parameters (such as pre-scan equilibration time, scan rate, temperature programming) were set and at least three buffer-buffer scans were performed before the sample was scanned. And DSC automated data analysis was conducted after experiments ran out. The DSC curves can read by Origin (Origin Pro 7.5).

### Construction of *P. aeruginosa pepP* Gene Mutant

To construct *pepP* mutants of *P. aeruginosa*, a two-step allelic exchange bacterial genome engineering strategy was employed (Hmelo et al., 2015; Sana et al., 2015). Briefly, in the first step of allelic exchange, the suicide vector pEX18Gm was integrated site-specifically into the chromosome of *P. aeruginosa* by homologous recombination, resulting in antibiotic-resistant single-crossover mutants. Then, in the second step of allelic exchange, a double crossover event occurred through a second homologous recombination, and the mutant was isolated using sucrose-mediated counter-selection. The corresponding mutants were finally identified by PCR and DNA sequencing. Specifically, PCRs were performed to amplify the target fragment sequences with upstream (800 bp) and downstream (800 bp) from *P. aeruginosa* chromosomal DNA, while the suicide plasmid pEX18Gm was linearized with gene-specific primers. The two PCR products were recombined with ClonExpress^®^ II One Step Cloning Kit (Vazyme), The resulting plasmid, pEX18-Gm-*pepP*, was then performed Site-directed mutagenesis or deletion. All these primers are listed in Supplementary Table S1. These vectors were then transformed into *E. coli* S17-1 and then mobilized into *P. aeruginosa* strains PA14 by conjugation, in order to transfer suicide plasmids from the *E. coli* donor S17-1 to the *P. aeruginosa* recipient PA14. Colonies were first screened using gentamicin resistance plates to get single-crossover mutants. And then the double-crossover mutants were screened by No-salt LB (NSLB) agar with 15% (wt/vol) sucrose. The *pepP* gene replacement mutant strain was further confirmed by PCR and DNA sequencing.

### Construction of Complementation Plasmid pRK415-*pepP*

To construct the complementation plasmid pRK415-*pepP*, PCR-amplified *pepP* was cloned into the EcoRI and HindIII sites of plasmid pRK415, giving rise to the plasmid pRK415- *pepP* (Lin et al., 2017). The recombinant plasmid and plasmid pRK415 were transformed into *E. coli* S17-1, respectively, and then mobilized into *P. aeruginosa* PA14-Δ*pepP* by conjugation to transfer pRK415-*pepP* from the S17-1 to PA14-Δ*pepP*. The PA14-Δ*pepP* strain carried pRK415-*pepP* or pRK415 plasmid were screened by *Pseudomonas* isolation agar (PIA) with 150 μg/ml tetracycline. For expression of PepP in the *P. aeruginosa* strains, the PA14-Δ*pepP* strain carried pRK415-*pepP* were induced by addition of 1 mM IPTG and then conducted cell invasion assays.

### HeLa Cell Invasion Assays

To enumerate bacteria internalized by HeLa cells to verify bacterium virulence, gentamicin survival assays were conducted with slight modifications (Chi et al., 1991). Briefly, mammalian HeLa cells (obtained from ATCC) were grown in DMEM medium, containing 10% (v/v) FBS (Gibco, Auckland, New Zealand) and 1 % antibiotics (penicillin and streptomycin) in 5% CO_2_ at 37°C. Suspension cultures of HeLa cells were seeded at 2–5 × 10^5^ cells per well in 12-well tissue culture plates for the overnight at 37°C. Cells were washed three times with PBS (pH 7.2) and changed to antibiotic-free medium immediately before infection. Cells were infected with exponential phase *P. aeruginosa* strains PA14 and mutant strains at a MOI of 10 for 1 h in 5% CO_2_ at 37°C. The cells were washed twice with PBS and incubated for an additional 1 h in DMEM medium containing 150 μg/ml of gentamicin in order to kill extracellular bacteria. The monolayers cells were then washed three times with PBS and lysed with 0.5% Triton X-100 for 10–20 min, and appropriate dilutions were plated on LB plates to determine the number of viable intracellular bacteria.

### Enzyme Activity Assays

The enzyme activity of wild type Pa-PepP and the mutants were spectrophotometrically determined using the quenched fluorescent substrate Lys (Abz) – Pro–Pro–pNA (synthesized by GL Biochem Shanghai Ltd., China) as the substrate. Prior to the assay, wild type Pa-PepP and the mutant enzyme are dialyzed with EDTA overnight to fully remove the pre-bound metal ions. And all enzyme preparations were freshly diluted with ice-cold 50 mM Tris (pH 8.5) and 100 mM NaCl and treated with MnCl_2_ for 10 min at 37°C. After addition of substrate, the final reaction conditions were 50 mM Tris (pH 8.5), 100 mM NaCl, 1 mM MnCl_2_, 0–250 μM substrate, and 20 nM Pa-PepP (final assay volume 100 μL). The assay was allowed to proceed for 5 min at 37°C in the Thermo Scientific Varioskan Flash plate reader. The appearance of fluorescent product (λex = 301 nm, λem = 410 nm) was monitored at 10 s intervals. Since the pure fluorescent product was not available to quantitate the changes in fluorescence, the kinetic results are presented as activities in relative fluorescence units. The kinetic parameters Km (Michaelis constant) for all the assays were obtained by fitting experimental data to the Michaelis-Menten equation by non-linear regression using the program OriginPro 7.5 (OriginLab Software). The data were showed in Supplementary Tables S2 and S3.

### Statistical Analysis

Statistical analysis was analyzed by 2-tailed Student’s *t*-test. In all statistical analysis, *P*-values < 0.05 were considered to be statistically significant.

## Accession Numbers

Atomic coordinates of the refined structures have been deposited in the Protein Data Bank (www.pdb.org) with the PDB code 5WZE.

## Author Contributions

C-TP performed the experiments and wrote this manuscript. LL planned experiments and analyzed data. C-CL conducted structural analysis. L-HH made modifications of the manuscript. TL performed the structure determination and refinement. Y-LS performed the HeLa cell invasion assays. CG planned the experiments. N-YW contributed the essential experiment materials. YX guided the experiments. Y-BZ constructed the *P. aeruginosa pepP* gene mutants. Y-JS also performed the HeLa cell invasion assays. QL made modifications of the manuscript and conducted the PCR experiments. L-TY guided the experiments and made modifications of the manuscript. RB wrote part of the paper and performed the drawing.

## Conflict of Interest Statement

The authors declare that the research was conducted in the absence of any commercial or financial relationships that could be construed as a potential conflict of interest.

## Supplementary Material

FIGURE S1 Relative activity of Pa-PepP in presence of different amount of Ca^2+^ **(A)** and Mg^2+^ **(B)**. All assays were performed at 37°C for 5 min in the presence of 50 mM Tris (pH 8.5), 100 mM NaCl, 50 μM Lyz(Abz)–Pro–Pro–pNA quenched fluorescent substrate, and 1 μg/mL^-1^ Pa-PepP that had been incubated with different concentrations of Ca^2+^ **(A)** and Mg^2+^ **(B)** at 37°C for 10 min. Each bar represents the mean of three independent measurements (SEM).

FIGURE S2 The differential scanning calorimetry (DSC) curves of wide type Pa-PepP and all the mutants. DSC assays were conducted to investigate the thermal stability of wild type Pa-PepP and the mutants. The enthalpy value and the melting temperature(Tm) value determined by DSC were showed in the DSC curves. As it showed in the DSC curves, all these protein samples shared the similar Tm value (66–68°C), with the only exceptional of Pa-PepP-E384A which has a acceptable shift of Tm value of 63°C.

FIGURE S3 Superposition of the monomer Pa-PepP and other APPros. **(A)** Superposition of the monomer Pa-PepP (red) with Ec-PepP (blue), hAPP1 (light gray), PfAPP (light blue), and CeAPP (light pink). The N domain and C domain of each APPro were circled with black frame and circle, respectively. **(B)** Superposition of the monomer Pa-PepP (red) with Ec-PepP (blue). The black circle refers to the non-conservative surface loop identified as an important motif in Pa-PepP’s function. **(C)** Schematic diagrams for the prokaryotic (right) and eukaryotic APPros dimers (left). N and C are labels for their N-terminal and C-terminal domains, respectively. And the plus N-terminal domain is labeled as N’.

FIGURE S4 Surface charge distribution of Pa-PepP and Ec-PepP. Surface charge distribution of Pa-PepP **(A)** and Ec-PepP **(B)** generated by CCP4mg (McCoy et al., 2007). Pa-PepP possesses more positively charged groups on its solvent-accessible area compared to Ec-PepP. The green circle refers to the predicted substrate entrance of Pa-PepP and Ec-PepP, and the entrance is also indicated by the red arrow near the black colored loop in the ribbon view of tetramer **(C)**.

## Abbreviations

DMEM: Dulbecco’s Modified Eagle’s Medium
FBS: fetal bovine serum
IPTG: isopropyl β-D-1-thiogalactopyranoside
LB: Luria–Bertani
MOI: multiplicity of infection
NCPSS: National Center for Protein Sciences Shanghai
OD600: optical cell densities at 600 nm
PBS: phosphate-buffered saline
*P. aeruginosa*: *Pseudomonas aeruginosa*
SDS–PAGE: sodium dodecyl sulfate–polyacrylamide gel electrophoresis
SSRF: Shanghai Synchrotron Radiation Facility
