# Supplementary material for: Structure–Function Relationship of Aminopeptidase P from Pseudomonas aeruginosa
Source: Front Microbiol. 2017 Dec 5;8:2385. doi: 10.3389/fmicb.2017.02385 (PMC5723419; doi:10.3389/fmicb.2017.02385)
Supplement: FIGURE S1 — Relative activity of Pa-PepP in presence of different amount of Ca2+ (A) and Mg2+ (B). All assays were performed at 37°C for 5 min in the presence of 50 mM Tris (pH 8.5), 100 mM NaCl, 50 μM Lyz(Abz)–Pro–Pro–pNA quenched fluorescent substrate, and 1 μg/mL-1 Pa-PepP that had been incubated with different concentrations of Ca2+ (A) and Mg2+ (B) at 37°C for 10 min. Each bar represents the mean of three independent measurements (SEM). [file Presentation_1.PDF]

Supplementary information

Title:

Structure-function relationship of Aminopeptidase P from *Pseudomonas aeruginosa*

Running Title:

Structural studies of Aminopeptidase P from P. aeruginosa

Cui-Ting Peng[1,2,#], Li Liu[1,2, #], Chang-Cheng Li[2], Li-Hui He[2], Tao Li[2], Ya-Lin Shen[2], Chao Gao[2], Ning-Yu Wang [2,3], Yong Xia[2], Yi-Bo Zhu[2], Ying-Jie Song[2], Qian Lei[2], Luo-Ting Yu[1,2,*], Rui Bao[2,*]

[1]Department of Pharmaceutical and Bioengineering, School of Chemical Engineering, Sichuan University. [2]Center of Infectious Diseases, State Key Laboratory of Biotherapy, West China Hospital, Sichuan University and Collaborative Innovation Center. [3]School of Life Science and Engineering, Southwest Jiaotong University.

[*] Address correspondence to Rui Bao, baorui@scu.edu.cn or Luo-Ting Yu, yuluot@scu.edu.cn

Supplementary Figure S1.

Relative activity of Pa-PepP in presence of different amount of Ca^{2+} (A) and Mg^{2+} (B).

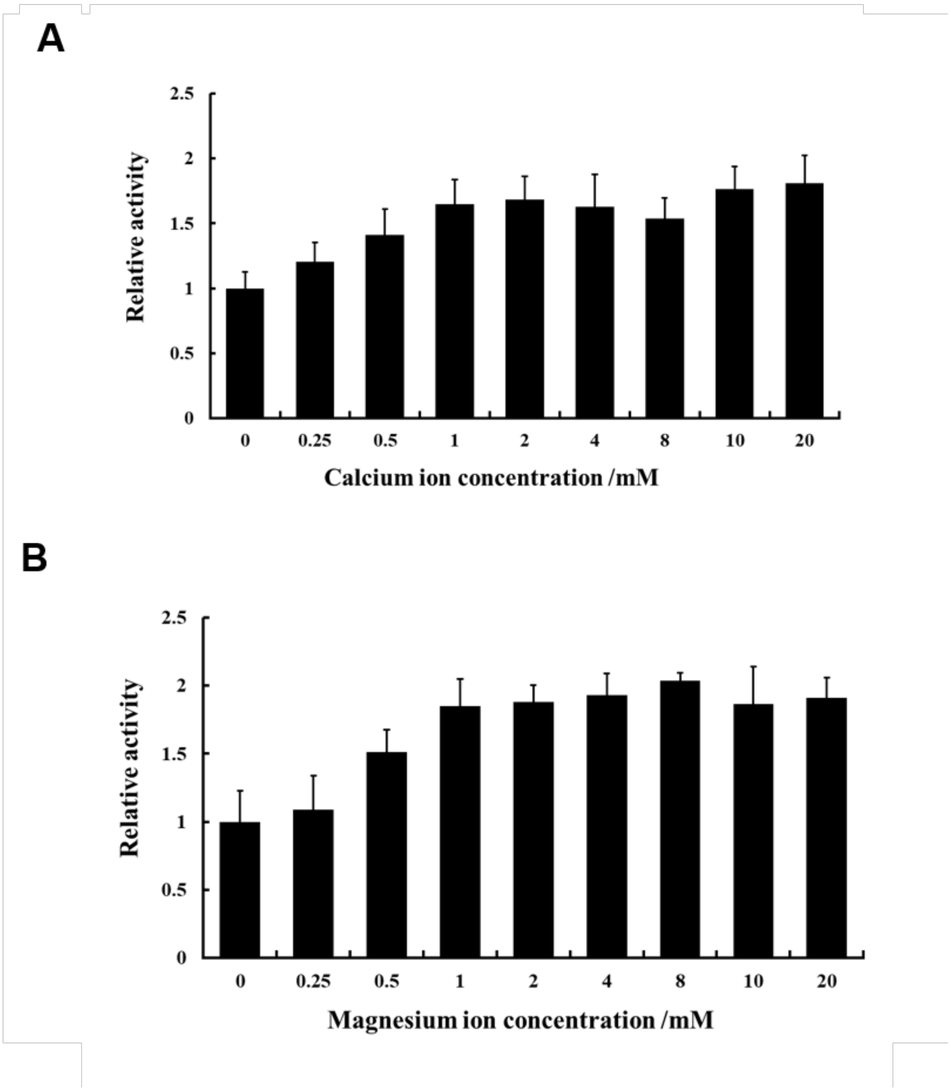

Supplementary Figure S2.

The DSC curves of wide type Pa-PepP and all the mutants.

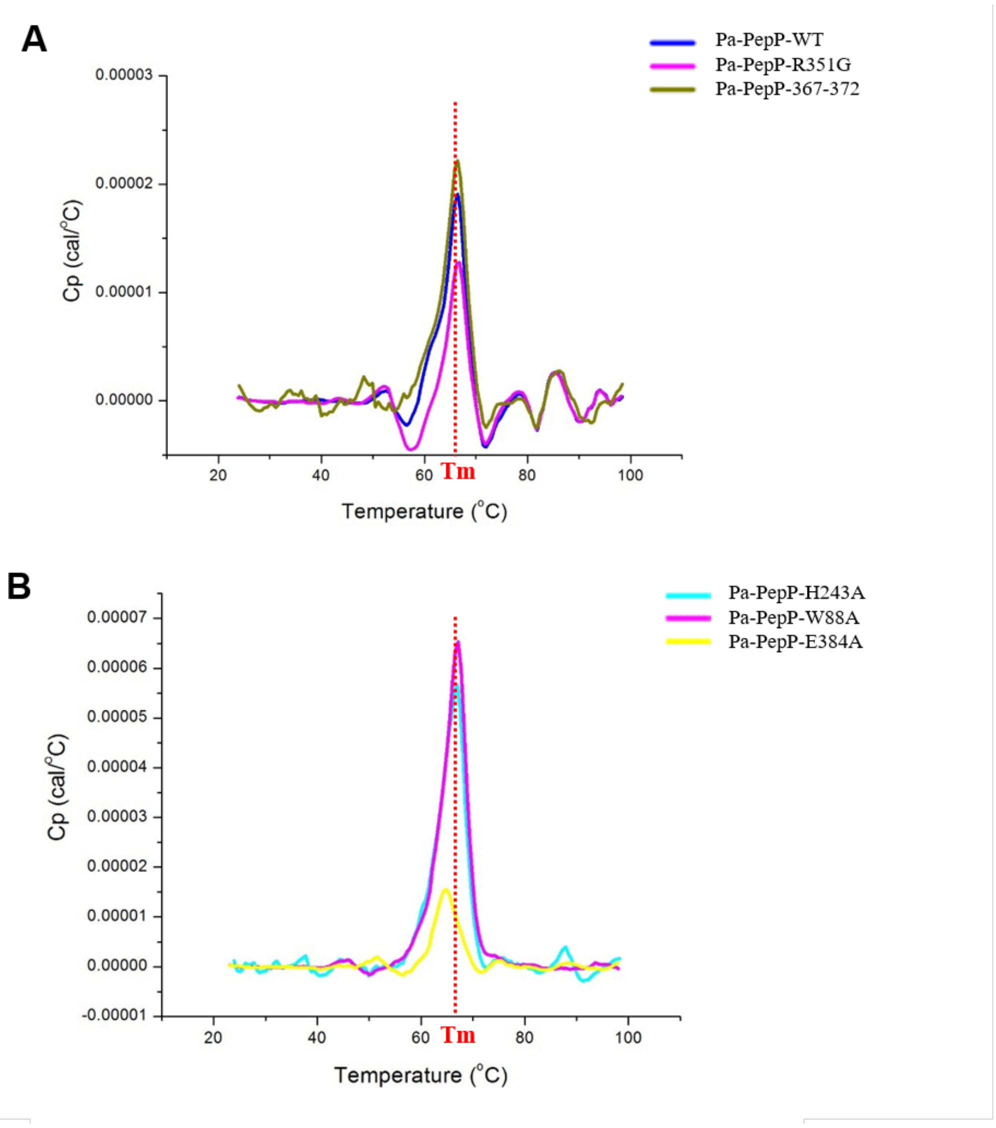

Supplementary Figure S3

. Superposition of the monomer Pa-PepP and other APPros.

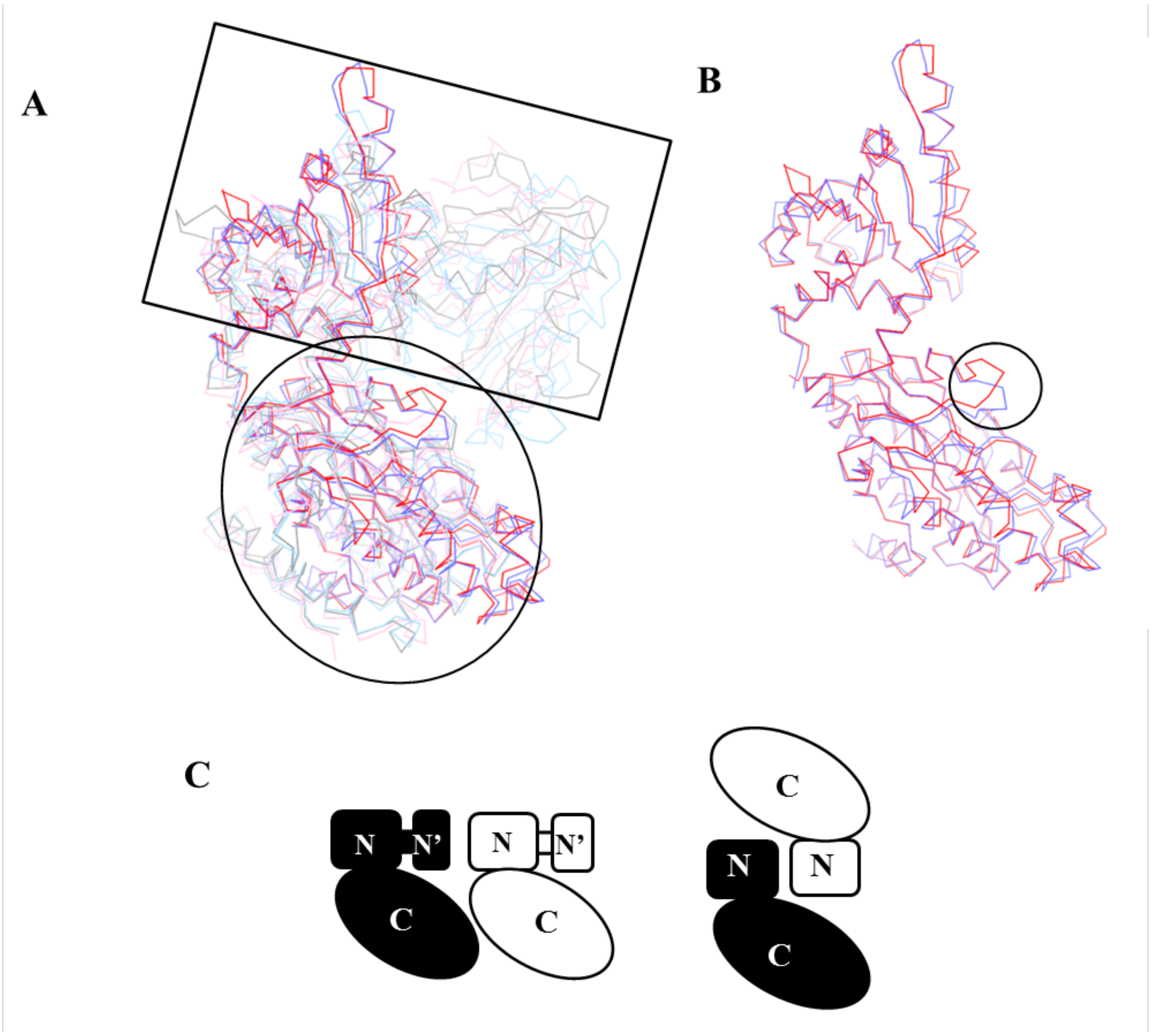

Supplementary Figure S4. Surface charge distribution of Pa-PepP and Ec-PepP.

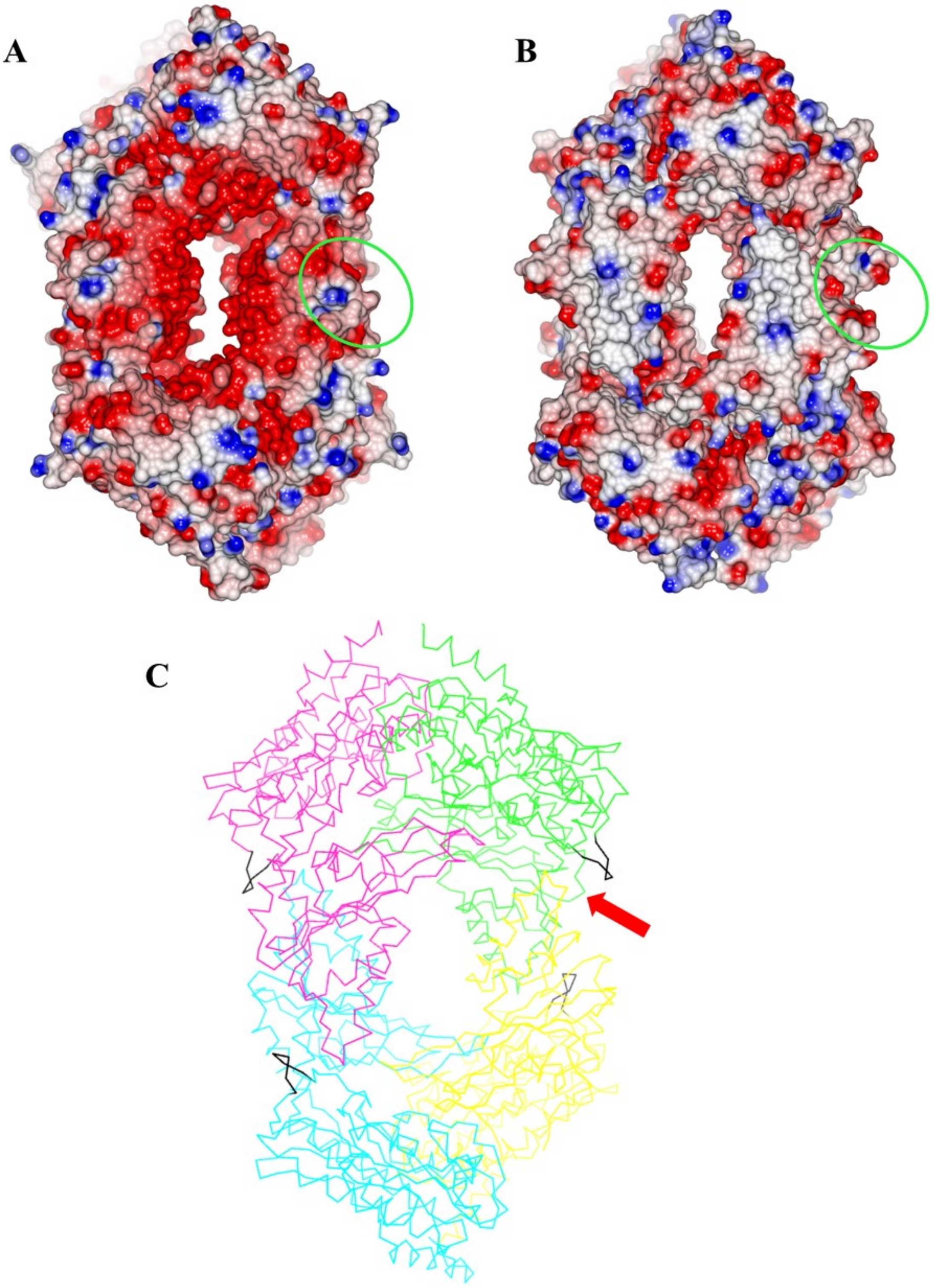

Supplementary Table S1. Related oligo primers used in this study

**Application**	**Template**	**oligos and sequences (5’ to 3’)**
**Protein expression**	PA14 genome	pET22b- *pepP*-FOR: TTAAGAAGGAGATATACATATGATGATCCGTATCCCCAAGTCG
		pET22b- *pepP* –REV: CTCAGTGGTGGTGGTGGTGGTGCTCGAGGGCTGCCTCGCTCTTG
	pET22b(+)	pET22b-line-FOR: CATATGTATATCTCCTTCTTAAAGTTAAA
		pET22b-line- REV: GATCTCAGTGGTGGTGGTGGTGGTGCTCGA
**Site-directed mutagenesis**	pET22b- *pepP*	Pa-PepP-R351G-FOR: TCTACATGCACGGCGCCGGCCACTGGCTGGGG
		Pa-PepP-R351G- REV: AAGCCTTGTAGGCCTCGTGGGCGATCAGCT
		Pa-PepP-367-372-FOR: GGTCAGGATCGCTCG CGGGTGCTCGAACCGGGC
		Pa-PepP-367-372- REV: CTGACCGTACTCGCCGACGTCGTGCACGTC
		Pa-PepP-E384A-FOR: GATGACCGTCGCCCCGGGCATCTACATCGCC
		Pa-PepP- E384A - REV: GCCATGCCCGGTTCGAGCACCCGCCATTCG
		Pa-PepP-H243A-FOR: CATCCTGGCCTACCGCGAGAACGATGCGGCGA
		Pa-PepP- H243A -REV: CAGGCGTTGCGTCCGGCGGCGACGATCGAG
		Pa-PepP-W88A - FOR: GAGCGTGAACTGGCCGACGGCCTGCGCGCC
		Pa-PepP- W88A -REV: CGGATCCCGCTCGCGGCAGAAAAGCACGTATTC
***Bacterial genome engineering**	PA14 genome	PEX18Gm- *pepP* -FOR: AACGACGGCCAGTGCCAAGCTTCAGCTGGTCGAACTTGGCCGTCA
		PEX18Gm- *pepP* - REV: TTCGAGCTCGGTACCCGGGGATCGCCGACCTGGCGGAAGGCGCCG
	PEX18Gm	PEX18Gm-line-FOR: TTCGAGCTCGGTACCCGGGGAT
		PEX18Gm-line-REV: AAGCTTGGCACTGGCCGTCGTT
	PEX18Gm- *pepP*	PA14-Δ*pepP*- FOR: GGTGACGATGAGCAAGGTCAACCTGGCGATCATCGGCGGGGGCCTGGTCG
		PA14-Δ*pepP*- REV: GCAGAAACCTCAGTGCAGTGAAGGCTTGGGAGCGGGCTCCAGCGGCTTGC

*The primes used in site-directed mutagenesis based on PA14 genome in Bacterial genome engineering, such as the PA14- Pa-PepP-R351G, PA14-Pa-PepP-R351G, PA14-Pa-PepP-367-372, are the same as those primers used in site-directed mutagenesis based on pET22b- *pepP.*

Supplementary Table S2. Kinetic parameters for the hydrolysis of the substrate by Pa-PepP in the presence of different metal ions[a].

Metal ion	Vm[b]	SD	Km(μM)[c]	SD	Relative Activity (%)	SD
Pa-PepP-Mn	1.77218	0.21062	133.62601	33.50577	100	11.8848
Pa-PepP-Ca	0.48875	0.02709	124.11271	14.29778	27.579027	1.528626
Pa-PepP-Mg	0.48078	0.0405	90.70354	17.5693	27.129298	2.285321
Pa-PepP-Ni					0	0
Pa-PepP-EDTA					0	0
Pa-PepP-Zn			`		0	0

[a]All assays were performed at 37 °C for 5 min in the presence of 50 mM Tris (pH 8.5), 100 mM NaCl, 0-250 μM Lyz(Abz)-Pro-Pro-pNA quenched fluorescent substrate, and 1 μg/mL-1 Pa-PepP that had been incubated with different metal ions at 37 °C for 10 min. Standard errors are shown (n=3). [b]Since the pure fluorescent product was not available to quantitate the changes in fluorescence, the kinetic results are presented as activities in relative fluorescence units. [c]The kinetic parameters Km (Michaelis constant) were determined using 0-250 μM Lys(Abz)-Pro-Pro-pNA and were obtained by fitting experimental data to the Michaelis-Menten equation by nonlinear regression using the program OriginPro 7.5 (OriginLab Software).

Supplementary Table S3. Kinetic parameters for the hydrolysis of the substrate by Pa-PepP and other mutants.

Mutant	Vm	SD	Km(μM)	SD	Relative Activity (%)	SD
Pa-PepP-WT	1.77218	0.21062	133.62601	33.50577	1	0.118
Pa-PepP-R351G	2.96256	0.09334	58.75224	5.4977	1.67	0.053
Pa-PepP-367-372	1.87042	0.13394	130.68535	19.92535	1.055	0.076
Pa-PepP-E384A	-	-	-	-	0.05	0
Pa-PepP-H243A	-	-	-	-	0.06	0
Pa-PepP-W88A	-	-	-	-	0.03	0
